# Psychophysiology of Monotonous Driving, Fatigue and Sleepiness in Train and Non-Professional Drivers: Driver Safety Implications

**DOI:** 10.3390/bs13100788

**Published:** 2023-09-22

**Authors:** Ty Lees, Taryn Chalmers, David Burton, Eugene Zilberg, Thomas Penzel, Sara Lal

**Affiliations:** 1 Edna Bennett Pierce Prevention Research Center, The Pennsylvania State University, University Park, PA 16802, USA; tlees@mclean.harvard.edu; 2 Center for Depression, Anxiety, and Stress Research, McLean Hospital, Belmont, MA 02478, USA; 3 Department of Psychiatry, Harvard Medical School, Boston, MA 02115, USA; 4 Medical Innovation Neuroscience Data-Analytics (MIND) Unit, TD School, University of Technology Sydney, Ultimo, NSW 2007, Australia; taryn.chalmers@uts.edu.au; 5 Compumedics Ltd., Melbourne, VIC 3067, Australia; dburton@compumedics.com.au (D.B.); ezilberg@compumedics.com.au (E.Z.); 6 Interdisciplinary Sleep Medicine Center, Charité Universitätsmedizin Berlin, 10117 Berlin, Germany; thomas.penzel@charite.de; 7 Honorary, School of Psychology, Faculty of Science, University of New South Wales, Sydney, NSW 2052, Australia; 8 Honorary School of Public Heath, University of Technology Sydney, Sydney, NSW 2007, Australia

**Keywords:** driving fatigue, drivers, electroencephalography, EEG, monotony

## Abstract

Fatigue and sleepiness are complex bodily states associated with monotony as well as physical and cognitive impairment, accidents, injury, and illness. Moreover, these states are often characteristic of professional driving. However, most existing work has focused on motor vehicle drivers, and research examining train drivers remains limited. As such, the present study psychophysiologically examined monotonous driving, fatigue, and sleepiness in a group of passenger train drivers and a group of non-professional drivers. Sixty-three train drivers and thirty non-professional drivers participated in the present study, which captured 32-lead electroencephalogram (EEG) data during a monotonous driving task. Fatigue and sleepiness were self-evaluated using the Pittsburgh Sleep Quality Index, the Epworth Sleepiness Scale, the Karolinksa Sleepiness Scale, and the Checklist of Individual Strength. Unexpectedly, fatigue and sleepiness scores did not significantly differ between the groups; however, train drivers generally scored lower than non-professional drivers, which may be indicative of individual and/or industry attempts to reduce fatigue. Across both groups, fatigue and sleepiness scores were negatively correlated with theta, alpha, and beta EEG variables clustered towards the fronto-central and temporal regions. Broadly, these associations may reflect a monotony-associated blunting of neural activity that is associated with a self-reported fatigue state.

## 1. Introduction

Fatigue is a non-discrete and complex bodily state [1], and its experience has been associated with impaired physical, cognitive, and psychomotor functions [2,3]. Furthermore, as a state, it is characterised by overwhelming tiredness/sleepiness and exhaustion, which, in turn, can have significant consequences, including an increased risk of physical accidents [4,5], injury, and illness [6,7]. Moreover, experiencing fatigue has been associated with declines in performance of the general population [8], as well as of industry professionals, including health workers [9,10], police officers [11], and most notably for the present work, drivers [12,13,14]. Lastly, prior to continuing through the introduction, it must be noted that a large body of previous work has used the terms fatigue and sleepiness interchangeably [15,16,17], mainly due to their extensive overlap in manifestation, and as a consequence, this paper also uses these two terms interchangeably.

As an industry, professional driving has been associated with a number of risk factors that may impact the wellbeing of its workers, including limited physical activity [18], cardiovascular and metabolic disease risk factors [19,20,21], and a generally increased disease burden [21,22]. Moreover, being a professional driver has also been associated with an increased incidence of heart disease [18,23,23] and stroke [24]. In addition, it is important to note that health status may impact driver safety [25], such that it could consequently contribute to increased accidents and incidents [26,27]. Operationally, professional driving is associated with long periods of minimal stimulation and lengthy periods of monotony. Importantly, the experience of said monotony has been associated with reductions in task efficiency and performance [28], greater psychological distress [29], and notably cardiac risk factors similar to those of being a professional driver [30].

Most important to the present work, experiencing the monotony characteristic of professional driving has been associated with the development and experience of driver fatigue, which has, in turn, been associated with a reduction in vigilance behaviour [31,32] and an increased likelihood of accidents [12,33,34]. Existing driver fatigue literature has evaluated both non-professional and professional drivers [35,36], with a primary focus on motor vehicle drivers, given that they are generally the most common mode of transport, and research in other driving modalities remains limited. Mass transport modalities (e.g., train driving) are part of a fundamental industry that provides a significant service to numerous other sectors and is staffed by people who may be at risk for experiencing beyond normal levels of fatigue and, as such, warrant attention [37,38]. Compared to the other driving professions, the specific requirements of train driving are unique, e.g., the timing of the journey (i.e., departure and arrival times) is set, which creates a time-pressured environment that contributes to the occurrence of reduced performance and potentially dangerous events [39]. The various impediments faced during the trip are generally predetermined, as are the length of the journey and the destination/s. In addition, this rigidity and predetermined nature, combined with the constantly required vigilance to identify and obey signals and signs that are often challenged by sighting restrictions (which also increase the risk of dangerous events [39]), often render train driving more inherently monotonous than other forms of driving, and this has subsequently been linked to greater fatigue experienced by the drivers [40]. It is also possible for this fatigue to carry over into the drivers’ lives away from their vehicle. In addition, several institutional factors within train driving, most notably schedule irregularities, have been reported to influence driving performance [41] and, as such, may further contribute to accident likelihood. Lastly, in terms of outreaching impact, trains, on average, move significantly faster and are larger and heavier than a standard motor vehicle. In addition, because of their size and weight, trains also have a slower braking time when compared to cars [42], and the combination of these characteristics drastically increases the potential impact of an accident.

The physiological changes associated with drivers experiencing fatigue have been examined in both professional and non-professional drivers [35,36]. Typically, these examinations either evaluate individual behavioural performance (i.e., number of errors, ability to adhere to signals/rules, etc.) on a driving task of interest or more commonly rely on self-reported data acquired from questionnaires like the Fatigue Severity Scale [43] or the Checklist of Individual Strength [44]. However, the physiological modelling of fatigue has more recently become an approach of interest. In this space, numerous physiological parameters, including, but not limited to, electroencephalography (EEG) [45,46,47,48], pupilometry [49], electrooculography [50], and multi-modal algorithms [51,52], have all been used to investigate the individual experience of fatigue and whether or not physiology can be used to predict experienced fatigue. Of these approaches, the present study chose to investigate EEG and its utility in examining monotonous driving and fatigue, as it has the most extensive literature base to work from and a sound theoretical mechanism. Mechanistically, previous research (and the current study) has invoked a classical arousal state interpretation of EEG activity, whereby as individuals experience a reduction in arousal (i.e., they descend through sleep stages), their neural activity shifts from the higher frequencies (i.e., beta and alpha) to slow-wave activity (i.e., delta and theta waves) [53]. As a body of work, the EEG in driver fatigue literature has generally found that as an individual’s fatigue and sleepiness increase, they exhibit an associated increase in slow-wave EEG activity (i.e., delta and theta brain activity) [37,54,55,56], as well as a decrease in fast-wave EEG activity (i.e., beta and gamma activity) [57,58,59]. Moreover, this literature demonstrates that examining physiological changes related to fatigue may provide insight beyond what behavioural and/or self-reported analyses deliver.

### The Present Study

Experiencing fatigue can have serious consequences for driving, not only in terms of the possibility of driving incidents and accidents but also with respect to the long-term health of the individual drivers. Train driving is no exception. Indeed, based on several characteristics inherent to the profession, its workers may be more susceptible to the detrimental effects of fatigue. However, there is at present a dearth of literature examining the experience of train drivers in relation to monotony and their fatigue and sleepiness. As such, the present study first sought to compare the self-reported fatigue and sleepiness between a group of Australian passenger train drivers and a group of non-professional drivers to elucidate any industry-specific effects. For this purpose, it was hypothesised that:

- When compared to their non-professional counterparts, the train driver sample group would self-report greater levels of fatigue and/or sleepiness.

This work then sought to examine how the experience of monotony during driving, as psychophysiologically indexed by EEG parameters, is associated with the self-reported fatigue and/or sleepiness of drivers. Based on existing literature and our previous work, it was hypothesised that:

- Increases in theta band activity and a concurrent reduction in alpha and beta band activity recorded during monotonous driving would be associated with increased self-reported fatigue and sleepiness for both train and non-professional drivers.

## 2. Materials and Methods

### 2.1. Participants

The present study recruited *n* = 63 passenger train drivers (54 male, 9 female) aged between 24 and 69 years (average 38.56 ± 9.96 years) who were employed full time by Sydney Trains to participate in the study session. Notably, as these drivers were all from the same government-operated service, they all operated within the same work schedule structure. Additionally, as the train drivers most likely also drive motor vehicles away from work, 30 non-professional drivers (as determined by possessing a driver’s licence and reporting at least 2 h of driving each week) aged between 20 and 65 years (14 male, 16 female; average 33.67 ± 13.61 years) also participated in the present study to provide insight into motor vehicle driving and function as a comparator for the effect of train driving.

Ethics approval for the current experimental protocol was provided by the University of Technology Sydney Human Research Ethics Committee. All recruited individuals were screened for any pre-existing medical conditions as well as medication and non-medical substance use known to effect physiological measurements using part one of the Lifestyle Appraisal Questionnaire (LAQ) [60]. As an additional requirement of the study’s ethics approval, prior to starting the experimental procedure below, the participants’ blood pressure (BP) values were assessed. If a participant exhibited blood pressure (BP) values of greater than 160 mmHg (systolic) and/or 100 mmHg (diastolic), they would be excluded from further participation, as such values place these individuals at an increased risk of cardiovascular events [61]. No recruited individual was excluded from participating in the present study.

All participants provided written informed consent prior to participating, and those participants who successfully completed the study were reimbursed a small sum of money that covered any out-of-pocket expenses incurred in participating in the present study. Finally, it is important to note that all study sessions were completed between 9:30 am and 3 pm in a controlled laboratory environment, and the train drivers completed their study session on a day where they were not working prior to or after the session.

### 2.2. Experimental Tasks and Procedures

To start the study protocol, participants initially rested seated for 5 min, after which three blood pressure measurements (with a two-minute rest between each reading) were recorded using an automated sphygmomanometer and averaged. This average value functioned as the aforementioned ethically required pre-study blood pressure measurement screen for each participant. If these blood pressure values were acceptable (i.e., <160 mmHg systolic and/or 100 mmHg diastolic), the participant then completed the remainder of the Lifestyle Appraisal Questionnaire [60] to self-report on various demographic variables. At this point in the protocol, participants also self-reported on their fatigue and sleepiness by completing one fatigue questionnaire, the Checklist of Individual Strength 20 (CIS20; [44]), and three sleepiness questionnaires: the Pittsburgh Sleep Quality Index (PSQI; [62]), the Epworth Sleepiness Scale (ESS; [63]), and the Karolinska Sleepiness Scale (KSS; [64]).

Upon completing the questionnaire battery, participants were set up for electrophysiological data collection. EEG data were digitised using a 1000 Hz sample rate combined with a 32-lead electrode montage supported by a SynAmp2 amplifier (Compumedics Ltd., Melbourne, Victoria, Australia) and the Scan software (Version 4.3, Compumedics Ltd., Melbourne, Victoria, Australia). Electrode placement was facilitated by using a 64-channel electrode QuikCap (Compumedics Ltd., Melbourne, Victoria, Australia), where the electrodes at the: Fp1, Fp2, F7, F3, Fz, F4, F8, FT7, FC3, FCz, FC4, FT8, T7, C3, Cz, C4, T8, TP7, CP3, CPz, CP4, TP8, P7, P3, Pz, P4, P8, O1, Oz, and O2 locations were filled with Signa Gel (Parker Laboratories Inc., Fairfield, NJ, USA). In addition, the ground and reference electrodes for the system were placed at position AFz and the vertex, respectively. Lastly, a bipolar vertical electrooculogram (EOG) was set up around the left eye, with one electrode placed above the eye in line with the pupil and one below.

Electroencephalographic data collection was conducted during a 10 min resting baseline phase. However, given the specific focus on driving in the present work, these data are not presented or analysed. Additionally, all participants also completed a 10 min active driving task, which varied between the two participant groups. The intended purpose of this task was not to induce fatigue in participants, but rather for participants to experience monotony while driving (i.e., a driving situation in which conditions stay the same and are therefore considered boring and largely unstimulating) and allow the recording of their brain activity during this condition.

More specifically, train driver participants were required to complete approximately 10 min of train driving using the first-person Trainz Classics driving simulator program (N3V Games, Queensland, Australia). Throughout this task, drivers were required to correctly adhere to any and all signals, if, and as they arose whilst they were moving the train between and stopping at stations. Non-professional driver participants undertook a near-equivalent 10 min recording in a car simulator that used a Logitech G7 racing wheel. In their task, non-professional drivers were required to safely (i.e., no crashes, obey signage if any, stay on the road, etc.) complete laps of a closed circuit (Gran Turismo 5) that was absent of any additional persons or computer-controlled cars. Notably, both sets of drivers were also advised that speed and the number of laps completed/stations stopped at were in no way relevant to the present study and that safely completing the driver was paramount. Moreover, both the train-driving and closed-circuit courses were located within similar virtual environments that were largely natural spaces with the occasional man-made features (e.g., a passing barricade/fence, or a small building). Finally, it is also worth mentioning that all subjects (both train drivers and non-professional drivers) were familiarised with their respective driving simulators and programs prior to the EEG recordings being obtained.

Following the completion of the 10 min task recording, electrophysiological data collection was concluded, and three post-study blood pressure measures were obtained as per the previous description, which concluded the experimental protocol. As a note, the blood pressure data is of no interest to the present study and was only captured as a requirement of the study’s ethics approval, and as such, it is not presented here.

### 2.3. Electroencephalography Data Processing

All raw recorded EEG data were filtered using a second-order Butterworth IIR bandpass filter with its high-pass and low-pass gates set at 0.5 and 50 Hz, respectively. Filtering was followed by the application of a Hanning window, after which the aligned-artefact average procedure [65,66] was applied to minimise eye movement artefacts. Following this first artefact removal procedure, the processed EEG data were manually screened for any egregiously noisy sections, which were subsequently removed. Post artefact removal, each EEG recording was sectioned into approximately 300 two-second epochs, and the spectral activity in the frequency bands of interest (theta [4–8 Hz], alpha [8–13 Hz], and beta [13–35 Hz]) [67] was calculated at each electrode for each individual epoch via a periodogram power spectral density estimate using Bartlett’s method [68,69].

After generating epoch-specific power values, the epoch-specific activity values within a frequency band for each individual electrode (i.e., 3 frequency bands per electrode) were evaluated for any outlying data, which were subsequently removed using a modified Z-score statistic [70] greater than or equal to 5, as calculated by the following equations:

(1) $$z=\frac{X-\tilde{x}}{MAD}$$

where X = epoch value; $\tilde{x}$ = median value; and MAD = median absolute deviation

Further, median absolute deviation was calculated using the following equation:

(2) $$MAD=\tilde{x}(\left|X_{i}-\tilde{x}\left(X_{i}\right)\right|)$$

where $\tilde{x}$ = Median and X_i_ = epoch value

After the removal of outlying epoch-specific activity values, a single activity value in each frequency band for each electrode location was derived by averaging all retained epoch values; this process led to 3 activity values per electrode for each recording. In addition, an overall average value was calculated for each frequency band by averaging the activity values at all electrode locations. After collating all of the processed EEG data, to remove extreme outlying values, the modified Z-score statistic previously applied was used to remove any outlying values at the population level (i.e., it was applied within the frequency band at each electrode to the sample level data); however, the removal threshold was set at a Z-score statistic greater than or equal to 10 [70]. Finally, it should be noted that the previously described Z-score was only used to identify outliers, and non-transformed data were entered into the analysis, as described below.

### 2.4. Data Analysis

The first aim of the present manuscript was to compare the self-reported fatigue and sleepiness between the two sample groups (i.e., train drivers and non-professional drivers), and this was achieved using independent sample *t*-tests. In addition, such tests were also used to compare demographic and driving-related data between the two sample groups. The second aim was to evaluate the neural impact of monotonous driving in both groups, which was achieved by calculating the partial Pearson’s correlation (controlling for participant age and body mass index [BMI]) among EEG parameters derived from data recorded during a monotonous driving task and self-reported fatigue and sleepiness questionnaire scores. This correlation analysis tested the null hypothesis of no correlation between parameters against the alternative that there is a non-zero correlation between said parameters.

All presented analyses were conducted with a statistical significance set at *p* < 0.05 using the STATISTICA software (Version 10, 2011, Statsoft Inc., Tulsa, OK, USA). Finally, it should be noted that while the correlations among the present train driver self-reported fatigue/sleepiness scores and EEG data have been previously reported [16], the aims, hypotheses, and combination of data utilised in the present research are unique and distinct from that work.

## 3. Results

Data from 93 individuals were included in the present analysis; 63 participants were professional train drivers (54 males; average age 38.56 ± 9.96 years), and 30 participants were non-professional drivers (14 males; average age 33.67 ± 13.61 years). As shown in Table 1, the total years of driving and weekly driving hours differed significantly between sample groups. Perhaps not unsurprisingly, train drivers reported being active for fewer years in their specific vehicle (*t* = 3.96, *p* < 0.001) but recorded a greater number of weekly driving hours (*t* = 12.75, *p* < 0.001).

### 3.1. Self-Reported Fatigue and Sleepiness

Regarding participant self-reported fatigue and sleepiness, each group’s average scores are presented in Table 2. With respect to between-group comparisons, the present analysis found no significant differences between the two sample groups for any of the utilised measures (*t*’s ≤ 0.53, *p*’s ≥ 0.31). As a note, despite the lack of significance, the non-professional driver group unexpectedly reported higher mean scores on three of four measures (the PSQI, CIS20, and KSS) than their train driver counterparts.

### 3.2. Fatigue/Sleepiness and Electroencephalography

As to the second aim of the present study, we examined the associations among theta, alpha, and beta band EEG parameters and self-reported fatigue and sleepiness scores so as to examine how the experience of monotony is linked to these constructs in both driver groups.

With respect to the self-reported PSQI score, the significant associations are presented in Table 3. For the non-professional driver group, the present analysis found central theta activity, frontal alpha activity, fronto-central, centro-parietal, and occipital beta activity to be significantly associated with PSQI score (*r*’s ≥ |0.48|, *p*’s ≤ 0.035). While in the train driver group, temporal and centro-parietal theta activity, frontal and fronto-temporal alpha activities, as well as fronto-temporal beta activity, were significantly correlated to self-reported PSQI score (*r*’s ≥ |0.32|, *p*’s ≤ 0.038). Almost across the board, these correlations were negative in nature, suggesting that increases in self-reported sleepiness are associated with a neural blunting when experiencing monotony.

Results for the second investigated sleepiness measure, the ESS, similarly suggest that sleepiness is associated with a neural blunting and the relevant correlations are presented in Table 4. For this measure, it was observed that the score of the non-professional driver group was significantly and general negatively correlated to centro-parietal and parietal alpha activity, and fronto-central and centro-parietal beta activity (*r*’s ≥ |0.45|, *p*’s ≤ 0.048). Interestingly, the results for the train driver group varied somewhat in terms of location, and their ESS score was only negatively correlated with frontal, fronto-temporal and parietal theta activity as well as frontal pole alpha activity (*r*’s ≥ |0.31|, *p*’s ≤ 0.048).

Lastly, for sleepiness, the associations among the KSS scores of both groups and their neural activity are presented in Table 5. Yet again, these were mostly negative in nature and indicative of a reduction in neural activity with monotony. More specifically, in the non-professional driver group, KSS score was significantly correlated with frontal theta activity, centro-parietal and parietal alpha activity, as well as fronto-central and fronto-temporal beta activity (*r*’s ≥ |0.48|, *p*’s ≤ 0.039). The correlation results for the train driver cohort were very similar, whereby the total KSS score was significantly correlated to reduced centro-parietal theta and alpha activity, as well as reduced frontal and parietal theta activity and temporo-parietal beta activity (*r*’s ≥ |0.33|, *p*’s ≤ 0.049).

Finally, moving to the specific fatigue measure, the CIS20, similar results to those of the sleepiness measures were observed (Table 6). More specifically, it was observed that the self-reported CIS20 score of non-professional drivers was generally negatively correlated with temporal and parietal theta activity, fronto-central and temporal alpha activity, and temporal beta activity (*r*’s ≥ |0.35|, *p*’s ≤ 0.042). In turn, for the train drivers, it was observed that their CIS20 was mostly negatively correlated with temporal and centro-parietal theta activity, frontal pole and occipital alpha activity, and fronto-temporal, temporo-parietal, and parietal beta activity (*r*’s ≥ |0.44|, *p*’s ≤ 0.049). As with the sleepiness results, these associations were mostly negative and suggest that as self-reported fatigue increases, the neural activity of both groups recorded during a monotonous task declines.

## 4. Discussion

In the present work, the impact of monotonous driving and self-reported fatigue and sleepiness were psychophysiologically examined in a group of Australian train drivers and a group of non-professional drivers. Overall, the two investigated sample groups reported surprisingly comparable fatigue and sleepiness scores, with no significant differences being recorded. Furthermore, when it came to neural activity recorded during monotonous driving, both groups again reported similar results, with associations between self-reported scores and reductions in theta, alpha, and beta activity being observed for both sample groups.

With respect to the demographic parameters of the two sample groups, they notably differed with respect to their BMI, where train drivers fell into the overweight category and non-professional drivers into the healthy category. This is not unsurprising for the train drivers, given their largely sedentary occupation. However, it does provide some concern, especially considering how professional driving has been associated with an increased incidence of heart disease and stroke [18,24], and that health status can impact driver safety [25] and rates of accident incidence [26,27]. In addition, because of this result, participant BMI was controlled for in the present correlation analysis.

The two groups also differed with respect to the distribution of the sexes within each participant group. In this sample, 85% of the train driver group identified as male, and only 47% of the non-professional drivers identified as male, marking a meaningful difference. However, while different, both distributions are reflective of their respective populations as a whole (i.e., train drivers and the general public). Nonetheless, men and women have been shown to experience various states differently, such as stress [71] and, more importantly, fatigue and sleepiness [72,73]. And so, the sex distribution of these groups presents a generalisability conundrum between using a representative population or one with an equal sex distribution, and this should be considered for the present results and future work.

Finally, for demographic data, as might be expected, the reported driving characteristics, i.e., years driving and weekly hours driven, varied significantly between the groups. Perhaps not unsurprisingly, the non-professional drivers reported a greater number of years of driving than their train-driving counterparts, a result most likely attributable to the ubiquity of motor vehicles. However, as we would expect, the non-professional drivers reported significantly fewer weekly driving hours than the train drivers. The variation in years driving is most poignant and was arguably most likely due to the modality-specific nature of the driving-related questions, e.g., years driving trains vs. years driving motor vehicles. Moving forward, capturing non-professional and professional driving experiences for all participants may enable a more holistic examination of fatigue/sleepiness in driving, particularly for professional drivers.

### 4.1. Self-Reported Fatigue and Sleepiness

Firstly, as previously reported [17], the self-reported scores of the train drivers varied with respect to previously published normative data [54,62,63,64,74]. The non-professional group self-reported similar variations, with scores above (PSQI, CIS20), below (ESS), and in line with (KSS) normative data. As was previously suggested for the train driver group [17], it is likely that this variability is representative of the respective subcomponents of fatigue and sleepiness that are imaged by each of the individual measures used in the present work, highlighting the importance of multi-modal and/or dimensional examinations of fatigue and sleepiness.

The direct comparison of self-reported fatigue and sleepiness scores between the two sample groups formed the first aim of the present study. In line with the monotonous nature of train driving, we hypothesised that the train driver sample group would experience greater fatigue than their non-professional counterparts. Interestingly, across the four separate comparisons of self-reported fatigue and sleepiness, the two sample groups did not report significantly different scores. That said, the non-professional group did report higher mean values for the KSS, PSQI (i.e., sleepiness), and CIS20 (i.e., fatigue), whereas the train driver group reported higher mean values for the ESS (i.e., sleepiness). Collectively, these comparisons are an important series of results, as train drivers are known to be more prone to experiencing fatigue due to the inherent context and demands of their profession [40].

There are a number of potential explanations for the lack of observed differences between the two groups. Firstly, as self-report questionnaires were utilised, the values obtained could quite possibly be an under-evaluation of the fatigue experienced, which certainly is not unheard of [75]. However, it should be recognised that this explanation applies to both groups. Although such an explanation is perhaps more applicable to the train drivers if their more regular experience of fatigue has shifted their perception of their experienced fatigue. Secondly, there is an argument to be made that fatigue-sensitive industries, in which professional driving is included, are becoming increasingly aware of and willing to manage fatigue and its impacts. To that end, Sydney trains, from which the present train drivers were recruited, have implemented a number of procedures to mitigate the impact of fatigue on their drivers. Further, at an individual level, it is possible that train drivers are becoming increasingly aware of their fatigue sensitivity and its implications and are actively attempting to combat it [76], as opposed to non-professional drivers, where fatigue mitigation is less crucial. As an alternative argument, it would be amiss to not consider the role of workplace culture and how a possible fear of employment repercussions (i.e., unwanted scheduling or routes, reduction in work hours or overtime availability, firing, etc.) for reporting fatigue, which has previously been reported [77], could also contribute to this lack of observed difference. While the present study cannot comment on such factors, such industry and workplace cultural factors are important and warrant attention in future research investigating industry-specific fatigue.

That said, if these results are associated with industry- or individual-level fatigue mitigation efforts, they may indicate promising change within the train driving industry. Indeed, it would be an interesting future direction to see if there are historical data that would enable an empirical examination of the outcome of these efforts. In a similar space, any future research investigating the prevalence and impact of fatigue would do well to capture data regarding whether or not participants try to combat fatigue and the specific methods employed.

### 4.2. Electroencephalography and Fatigue and Sleepiness

The second aim of the present work was to examine the neural impacts of experiencing monotony while driving and whether EEG data recorded during this task correlated with self-reported fatigue and sleepiness. From an arousal state framework, we hypothesised that increases in theta activity and a concurrent reduction in alpha and beta frequency band activity would be associated with increased self-reported fatigue and sleepiness for both driver groups.

In the present correlation analysis, it was observed that for the train driver group, increases in self-reported fatigue and sleepiness were associated with various changes in the investigated EEG band activity, the majority of which can be summarised as reductions in fronto-temporal and centro-parietal theta activity, frontal and fronto-temporal alpha activity, and fronto-temporal and parietal beta activity. Similar results were observed in the non-professional driver group, with temporal and parietal theta parameters, frontocentral, centro-parietal, and temporal alpha parameters, and fronto-central, centro-parietal, and parieto-occipital beta parameters being associated with self-reported scores.

For the most part, these associations are in line with our hypothesis, whereby, as fatigue and sleepiness scores increase, brain activity, particularly in the alpha and beta bands, declines [13,47]. Furthermore, the heavy implication of reductions in fronto-central parameters and the role of these regions in executive and higher-order functions [78,79] certainly provide strength to the relationships identified and perhaps suggest a localisation of the effect of monotony. Beyond this, the implication of reduced theta activity in both groups is somewhat unexpected. The arousal state framework used would suggest that theta activity should increase as an individual experiences fatigue, and the absence of this result perhaps points towards a more general neural blunting as the result of experiencing monotony. Exploring within-person time-courses of monotony and related neural activity over longer simulator tasks (i.e., comparing activity values for multiple successive 10 min blocks) could be a useful way to further evaluate this reduction in slow-wave activity. As an additional future thought related to neural blunting, it could be interesting to simultaneously examine brainstem activity during a driving task (e.g., using brain-stem auditory potentials) to evaluate if an equivalent or correlated reduction in reticular-activating system activity is present due to its implication in forebrain awakening [80,81].

Collectively, these results not only provide insight into the experience of monotony during driving and how this relates to self-reported fatigue, but also have the possibility of functioning as a basis on which researchers could develop and test physiologically grounded algorithms for fatigue detection. That said, it should be noted that the present analysis identified a few positive relationships between self-reported scores and EEG parameters (e.g., frontal theta and temporoparietal beta). While these appear to be outliers among all the results, these associations do warrant further attention, especially to consider and evaluate if they are specific to a subcomponent of fatigue/sleepiness, individual differences related to the drivers, or are more broadly replicable.

### 4.3. Limitations and Future Directions

As with any piece of work, there are limitations to the present study. While the sample sizes of both the train driver and non-professional driver groups are adequate for the present analysis and comparable to those of other similar studies (see Table 1 from [82]), they could be improved. Indeed, bringing them in line with the sample sizes of questionnaire-based research (e.g., [83]) would certainly improve the generalisability of the present work and allow for a more direct comparison across the literature. Moreover, research has shown that numerous factors, including institutional (e.g., schedule regularity and structure) and individual (e.g., outside work issues contributing to fatigue), have been reported to impact driving performance and the health of drivers [41]. As such, future research should consider evaluating drivers’ perceptions of these factors in their analysis and not just relying on self-reported fatigue and sleepiness scores.

In addition, data were captured at only a single time point and had all drivers complete a single driving task. While this allows for an initial insight into monotony and fatigue/sleepiness, time of day is very likely to be an important co-varying factor in the analysis of fatigue, especially as night driving has been associated with variations in markers of sleepiness and fatigue when compared to daytime driving [84,85,86]. And so, future research would do well to capture data at multiple time points throughout this cycle, as this would allow for a more extensive and robust examination of fatigue, in particular a within-person analysis comparing individual differences between the time points. This would also present the opportunity to control for any effects of circadian rhythms and diurnal variations in EEG parameters.

With respect to the driving tasks used in the present work, simulators provide a safe and accessible means to evaluate driver fatigue. However, they acutely lack consequences for driver error, which may modulate a driver’s engagement with the task and the observed results. As such, if resources are available, it would be salient for controlled conditions, closed circuits, and/or other more realistic testing to be utilised in future driving tasks over or alongside the present simulator tasks. In addition, using these conditions or simulators to test across multiple simulated environments to evaluate differences in sighting restrictions, time pressure, and interactions with stations and controllers [39] may also help further understand how monotony and driver fatigue intersect.

Moreover, to further expand the previous point of testing across multiple time points within the day, future studies should also consider extending the length of the driving task. This could be either to a fixed time point, e.g., an hour, or to failure, i.e., the presence of physical indicators of fatigue (e.g., head nodding, eyes closing, etc.), such that the experienced monotony of the task would push towards actively inducing changes in state-level fatigue, which may prove an insightful manipulation.

In addition, as comparisons between average train driving and motor vehicle driving experience will inherently be lopsided, and this may obscure the true nature of the results, future research would do well to find an intelligent means to incorporate driving experience across modalities. Options for this may include separately collecting personal and professional driving experience data for all participants or even having all participants complete both simulated tasks (i.e., non-professional drivers complete the train driving task and train-drivers complete the car driving task), such that the effects of monotony between groups can be more directly compared.

Lastly, as noted throughout the discussion, multi-modal examinations appear to provide the most insight into the experience of monotony and fatigue. As such, future psychophysiological work may wish to incorporate not only behavioural and physiological data but also multiple physiological modalities such as HRV, EDA, etc. Furthermore, such work may also consider using non-traditional approaches to processing captured EEG and/or physiological data, e.g., principal component analysis and decomposition, neural decoding of ERP signals, etc., as such steps can often provide interesting descriptive features that are not discernible by traditional spectral analyses. Moreover, including EEG coherence, synchronicity, and oscillatory parameters and/or non-traditional EEG variables [87] and/or interaction variables, e.g., power spectral ratios or neural-cardiac coupling values [88], in future analyses also warrants future attention, as it is possible that such parameters may better represent monotony and fatigue [51,89]. Similarly, if research is more interested in acute impairments and/or the occurrence of driving errors (e.g., missed signals), an analysis of event-locked EEG data reminiscent of a more traditional ERP approach could also prove valuable. However, researchers should be conscious of the psychometric properties of ERPs and the traditional need for repeated measures. However, it is worth noting that single-trial ERP analysis methods are improving and becoming more ubiquitous.

## 5. Conclusions

The present study conducted a psychophysiological comparison of the self-reported fatigue and sleepiness in a group of Australian train drivers and a group of non-professional drivers. Interestingly, no significant differences in the self-reported fatigue and sleepiness of the two groups were observed. Although somewhat unexpected, the train driving group reported lower scores, suggesting that they are on average less fatigued than their non-professional counterparts. It may be that this lack of between-group differences is related to individual attempts by the train drivers or operator (i.e., Sydney Trains) to take specific actions or initiatives to combat fatigue and could indicate promising change within the industry. However, it is also possible that a fear of possible employment repercussions for reporting fatigue might also be contributing to this lack of difference.

Moreover, the present work also investigated the neural impact of monotonous driving in these two groups using EEG and largely found results that were as expected. Across all of the investigated frequency bands, it was generally found that as self-reported fatigue and sleepiness scores increased, the recorded neural activity declined, with some potentially task-specific variation in these changes between the two groups. These associations appear to reflect a monotony-associated blunting of neural activity that is associated with self-reported fatigue states and may provide a foundation from which fatigue researchers could develop and test physiologically grounded algorithms for fatigue detection.

## Figures and Tables

**Table 1:** Demographic data of and intergroup comparison between the two study sample groups.

Variable	Group	*n*	Mean ± SD	*t*	*p*
Age (years)	TD	63	38.56 ± 9.96	1.93	0.06
	NPD	30	33.67 ± 13.61		
BMI (kg/m^2^)	TD	63	29.13 ± 5.53	−4.64	<0.001 *
	NPD	30	24.68 ± 3.40		
Years Driving	TD	63	7.43 ± 6.02	3.96	<0.001 *
	NPD	30	16.90 ± 13.64		
Weekly driving hours	TD	63	32.21 ± 8.53	12.75	<0.001 *
	NPD	30	8.83 ± 7.29		
Lifestyle Risk Factors (LAQ P1)	TD	63	15.79 ± 7.36	0.49	0.63
	NPD	30	15.00 ± 7.32		

Note: *t* and *p* values are provided for the intergroup comparisons for each demographic variable. BMI = Body mass index; LAQ = Lifestyle Appraisal Questionnaire; n = Sample size; NPD = Non-professional drivers; P1 = Part one; SD = Standard deviation; TD = Train drivers * = Statistical significance.

**Table 2:** Self-reported fatigue scores of the train driver and non-professional driver sample groups.

Variable	Group	Mean ± SD	*t*	*p*
PSQI Global	TD	6.43 ± 3.09	−0.20	0.84
	NPD	6.60 ± 4.83		
CIS20 Global	TD	59.78 ± 20.23	−1.03	0.31
	NPD	64.47 ± 20.37		
ESS	TD	4.89 ± 3.85	0.53	0.60
	NPD	4.47 ± 3.09		
KSS	TD	3.56 ± 1.63	−0.20	0.84
	NPD	3.63 ± 1.79		

Note: *t* and *p* values are provided for the intergroup comparison of each fatigue and sleepiness variable. CIS20 = Checklist of Individual Strength; ESS = Epworth Sleepiness Scale; KSS = Karolinska Sleepiness Scale; n = Sample size; NPD = Non-professional driver; PSQI = Pittsburgh Sleep Quality Index; SD = Standard Deviation; TD = Train Driver.

**Table 3:** Associations between the total PSQI score of both train and non-professional drivers and various spectral electroencephalography parameters.

Sample Group	EEG Variables	*n*	*r*	*p*
Train Drivers	T7-θ	43	−0.38	0.013
	CPz–θ	39	−0.33	0.038
	Fp1–α	42	−0.33	0.033
	Fz-α	42	−0.32	0.038
	FT7–α	42	−0.34	0.029
	FT7-β	40	−0.35	0.025
	FT8-β	36	−0.37	0.028
Non-professional drivers	C3-θ	22	−0.51	0.015
	F8-α	17	−0.51	0.035
	F4–β	19	−0.49	0.034
	FC3–β	16	−0.59	0.017
	CP4–β	20	−0.50	0.026
	O1–β	29	0.48	0.008
	Mean-β	29	0.58	0.001

Note: Non-significant correlations are not presented for clarity. C = Central; F = Frontal; Fp = Frontal Pole; n = Sample size; O = Occipital; P = Parietal; PSQI = Pittsburgh Sleep Quality Index; T = Temporal; z = Midline; α = Alpha; β = Beta; θ = Theta.

**Table 4:** Associations between the total ESS score of both train and non-professional drivers and various spectral electroencephalography parameters.

Sample Group	EEG Variable	*n*	*r*	*p*
Train Drivers	F7–θ	45	−0.33	0.026
	F8–θ	40	−0.32	0.045
	FT7-θ	44	−0.32	0.032
	P7-θ	36	−0.36	0.029
	Fp1–α	41	−0.31	0.048
Non-professional drivers	CP3–α	22	−0.49	0.022
	P3–α	23	−0.51	0.012
	P4–α	25	−0.50	0.011
	FC3–β	16	−0.57	0.022
	CP3–β	20	−0.45	0.048
	CP4–β	20	−0.54	0.014

Note: Non-significant correlations are not presented for clarity. ESS = Epworth Sleepiness Scale; F = Frontal; Fp = Frontal pole; n = Sample size; P = Parietal; T = Temporal; α = Alpha; β = Beta; θ = Theta.

**Table 5:** Associations between the total KSS score of both train and non-professional drivers and various spectral electroencephalography parameters.

Sample Group	EEG Variable	*n*	*r*	*p*
Train Drivers	F3-θ	37	0.35	0.033
	CP3-θ	35	−0.40	0.018
	P3-θ	39	−0.33	0.039
	P8-θ	38	−0.35	0.032
	CP3-α	35	−0.35	0.041
	TP8-β	34	0.34	0.049
Non-professional drivers	F3–θ	19	0.59	0.008
	CP3–α	22	−0.48	0.027
	P3–α	23	−0.58	0.004
	FC3–β	16	−0.51	0.039
	FT8–β	20	−0.50	0.026

Note: Non-significant correlations are not presented for clarity. C = Central; F = Frontal; KSS = Karolinksa Sleepiness Scale; *n* = Sample size; P = Parietal; T = Temporal; α = Alpha; β = Beta; θ = Theta.

**Table 6:** Associations between the total CIS20 score of both train and non-professional drivers and various spectral electroencephalography parameters.

Sample Group	EEG Variable	*n*	*r*	*p*
Train Drivers	T7–θ	43	−0.43	0.003
	CPz-θ	49	−0.35	0.029
	Fp1–α	42	−0.35	0.022
	Oz-α	30	−0.37	0.042
	FT8-β	36	−0.43	0.009
	TP8–β	35	0.40	0.016
	P3–β	35	−0.39	0.022
	P4-β	36	−0.39	0.017
Non-professional drivers	T8-θ	18	−0.55	0.018
	P7–θ	23	−0.44	0.035
	FC4-α	18	0.47	0049
	T8-α	17	−0.50	0.041
	T8–β	21	−0.54	0.012

Note: Non-significant correlations are not presented for clarity. C = Central; CIS20 = Checklist of Individual Strength; F = Frontal; Fp = Frontal pole; n = Sample size; O = Occipital; P = Parietal; T = Temporal; z = Midline; α = Alpha; β = Beta; θ = Theta.

## Data Availability

The data that support the findings are not publicly available due to privacy or ethical restrictions and are available on request from the corresponding author.
